# Can lead damage be ruled out using defibrillation threshold testing in patients with very high‐impedance shock leads?

**DOI:** 10.1002/joa3.70014

**Published:** 2025-02-12

**Authors:** Masataka Narita, Yoshifumi Ikeda, Hitoshi Mori, Ritsushi Kato, Kazuo Matsumoto

**Affiliations:** ^1^ Department of Cardiology Saitama Medical University, International Medical Center Saitama Japan

**Keywords:** defibrillation threshold testing, low‐voltage subthreshold measurement, transvenous implantable cardioverter defibrillator, true shock impedance, very high shock impedance lead

## Abstract

This is the first report describing a shock lead whose functionality can be assessed using TSI measurement, even in cases where shock impedances derived from LVSM exceed 200 Ω. However, the lead exhibited high shock impedance after the DFT test, highlighting the need to monitor it closely.

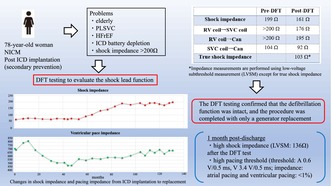

## 1 INTRODUCTION

The shock impedance of a transvenous implantable cardioverter defibrillator (ICD) can be measured in two main ways: high‐voltage shock impedance (HVSI), which involves using a high‐energy shock, and low‐voltage subthreshold measurement (LVSM). LVSM applies low voltages at pain‐free threshold levels to approximate the true shock impedance (TSI), ideally requiring HVSI measurement.^1^ However, HVSI measurement is invasive and is rarely used today. The transvenous ICD lead produced by Boston Scientific (Marlborough, MA, USA) uses an integrated bipolar configuration for sensing, and it uses LVSM to evaluate the shock impedance. This report recorded a maximum impedance of 167 Ω.^2^

Generally, a shock impedance exceeding 200 Ω indicates potential lead failure, which may necessitate lead replacement or the implantation of an additional lead. This is the first reported case of an Endotak Reliance 0296 lead (Boston Scientific) showing a progressive increase in shock impedance over time, ultimately exceeding 200 Ω and demonstrating a significant discrepancy between LVSM and TSI.

## 2 CASE REPORT

**FIGURE 1:**
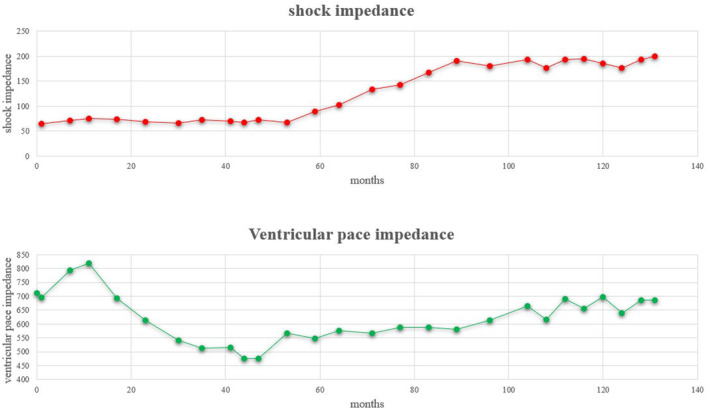
Course of shock impedance and ventricular pacing impedance over time following ICD implantation.

A 78‐year‐old woman was admitted for ICD replacement and lead management because of transvenous ICD battery depletion and very high shock impedance. She had been transported to our hospital 11 years earlier following a cardiac arrest caused by ventricular fibrillation, at which time an ICD (INCEPTA F162: Boston Scientific, Marlborough, MA, USA) was implanted for secondary prevention. On admission, low left ventricular function was observed, leading to a diagnosis of dilated cardiomyopathy. A persistent left superior vena cava was identified during the initial implantation, leading to the placement of the device on the right side. Defibrillation threshold (DFT) testing was not performed during implantation, and the initial shock impedance measured by LVSM was 65 Ω. Over time, shock impedance gradually increased during outpatient follow‐up, and the battery became depleted. Eventually, the shock impedance exceeded 200 Ω (Figure 1). Several physical exercise maneuvers were carefully performed, but no lead noise was detected. The patient was hospitalized for ICD replacement, with consideration given to adding a shock lead. The results of the pacemaker check before discharge were as follows: amplitude: A 2.2 mV, V 25 mV; threshold: A 0.6 V/0.5 ms, V 3.4 V/0.5 ms; impedance: A 543 Ω, V 685 Ω, high voltage >200 Ω; atrial pacing and ventricular pacing: <1%.

Treatment options included lead extraction, placement of a new lead, and DFT testing to evaluate the shock lead function. Lead extraction was deemed risky because of the patient's advanced age, the 11‐year‐old lead, and the presence of a superior vena cava coil. There was a potential obstruction in the right subclavian vein, and placing an additional lead was also challenging because of the persistent cleft superior vena cava, which made lead placement through the left subclavian vein difficult. DFT testing carries a relatively low risk, and if the TSI is acceptable, the battery can be replaced without needing to replace the lead, reducing potential risks. After a multidisciplinary conference that included input from the manufacturers, the decision was made to obtain informed consent from the patient and proceed with the DFT testing as the initial approach. We planned to proceed with device replacement if no shock lead failure was detected and to re‐evaluate the need for more invasive interventions if any issues arose.

**FIGURE 2:**
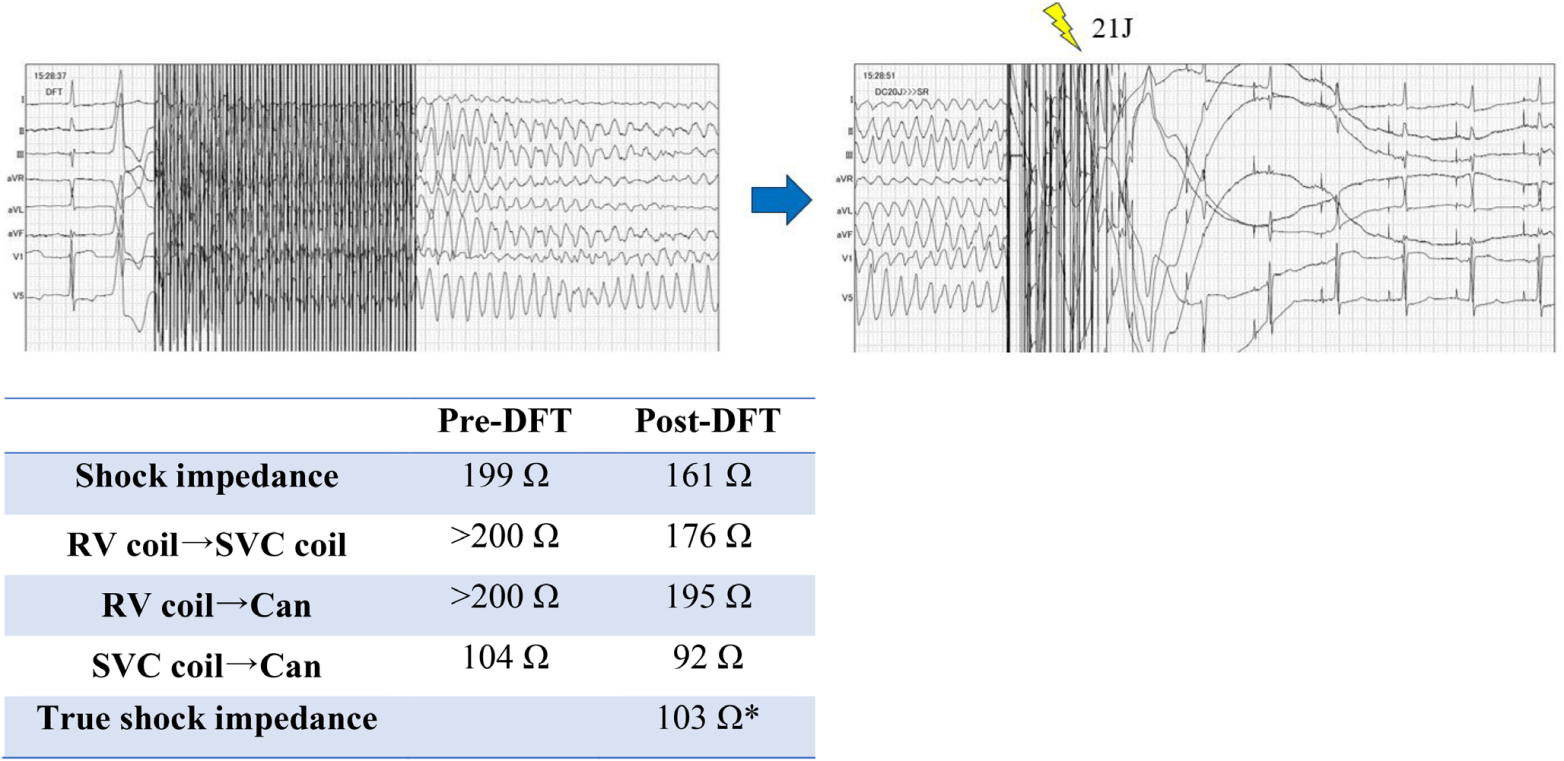
Electrocardiogram during the DFT, along with shock impedance measurements and true shock impedance before and after the test. *Impedance measurements are performed using low‐voltage subthreshold measurement except for TSI. DFT, defibrillation threshold test; RV, right ventricular; SVC, superior vena cava; Can, device casing.

Ventricular fibrillation was induced and successfully terminated with a 21 J shock from the ICD, with TSI recorded at 103 Ω (Figure 2). The DFT testing confirmed that the defibrillation function was intact, and the procedure was completed with only a generator replacement. At the outpatient follow‐up visit 1‐month post‐discharge, shock impedance measured using LVSM was 136 Ω.

## 3 DISCUSSION

DFT testing is generally considered less effective and is not routinely performed today.^3^ Integrated bipolar shock leads lack a proximal ring electrode, with the distal shocking coil serving as the anode for both pacing and sensing. In this case, the increase in pacing impedance was gradual, unlike the typically sharp rise seen with lead damage. If lead damage were present, both impedances would be expected to increase significantly, suggesting that lead function may still be preserved.

Monkhouse et al. have attributed the increase in lead impedance to environmental stress cracking as a cause of lead damage.^2^ Environmental stress cracking results from mechanical stress associated with heartbeat, which weakens the surface structure and causes brittle microcracks. In this case, the lead fraction was considered improbable due to the absence of observed noise.^4^ Performing biological or invasive procedures to examine capsular tissue is challenging, and DFT testing provides an alternative for assessing the need for lead replacement or additional leads.

The ICD shock lead Endotak Reliance 0296 is covered with GORE expanded polytetrafluoroethylene (ePTFE) to prevent tissue ingrowth around and between the shock coils.^5^ This shock lead is known to have the potential to develop calcification approximately 7 years post‐implantation.^5^ Calcification can lead to high impedance, which may interfere with defibrillation and pacing.

Furthermore, when GORE ePTFE‐coated coils were introduced, it was noted that improper implantation could damage the coating and expose the coil. Monkhouse et al. demonstrated that evaluating high shock impedance dysfunction in Boston Scientific ICDs can be complex, but TSI can be accurately measured through DFT testing.^2^ Their flow diagram recommends that patients with high‐voltage impedance measurements be monitored, with repeat shock delivery if the true high‐voltage impedance falls within the normal range.^2^

Monkhouse et al. suggested the theory is ionic build up around the RV coil. Ionic build up that is dissipated with ICD shock may prove a reason as to why the LVSM transiently drops post‐shock before increasing once more.^2^ In our opinion, defibrillation may have an organic effect on the fibers surrounding the shock coil.

Based on the above considerations and findings from this paper, physical structural changes in this ICD lead coated with GORE ePTFE likely contributed to the observed high impedance measured by LVSM.

The MAUDE Adverse Event Report about ENDOTAK RELIANCE G reported that high‐impedance shocks out‐of‐range occurred in the lead, but no clinical adverse events happened.^6^ However, leads with high pacing impedances and thresholds in pacemaker‐dependent patients may warrant replacement to prevent pacing failure.^5^

This is the first report describing a shock lead whose functionality can be assessed using TSI measurement at the time of battery replacement, even in cases where shock impedances derived from LVSM exceed 200 Ω. In this case, the lead exhibited high shock impedance after the DFT test and a high pacing threshold, highlighting the need to consider encapsulation effects and monitor it closely. It is also crucial to fully recognize the limitations of this otherwise valuable lead, including potential inaccuracies in LVSM and lead fragility.

## 4 CONCLUSION

DFT testing was performed on a shock lead with very high shock impedance on LVSM, allowing for appropriate evaluation and management of the lead condition.

## AUTHOR CONTRIBUTIONS

Y.I and R.K planned the treatment method; N.M drafted the manuscript; H.M revised the manuscript; and K.M supervised the study.

## FUNDING INFORMATION

The auathors have nothing to report.

## CONFLICT OF INTEREST STATEMENT

Y.I. received an honorarium from Medtronic Japan, Biotronik Japan, and Boston Scientific Japan as a speaker and a procedure trainer. All other authors report no conflicts of interest.
